# Exploring the Chemical Profile, Antioxidants, and Anti‐Diabetic Properties of Coffee Beans From Selected East African Countries: A Comparative In Vitro and Computational Study

**DOI:** 10.1002/fsn3.70527

**Published:** 2025-07-14

**Authors:** Almahi I. Mohamed, Ochuko L. Erukainure, Huda Ismail, Md. Shahidul Islam

**Affiliations:** ^1^ Department of Biochemistry, School of Life Sciences University of KwaZulu‐Natal Durban South Africa; ^2^ Department of Microbiology, School of Life Sciences University of KwaZulu‐Natal Durban South Africa

**Keywords:** antidiabetic, antioxidants, caffeine, coffee beans, phenolic acids, Type 2 diabetes

## Abstract

East Africa, the origin of coffee beans, is renowned for its coffee's cultural significance and potential health benefits, particularly in mitigating metabolic disorders and their related complications. Despite this, studies that comprehensively evaluate their pharmacological potential remain limited. This study investigated the antioxidant and antidiabetic properties of coffee beans from Uganda, Burundi, and Tanzania using in vitro and computational methods. Antioxidant activities were evaluated via DPPH, FRAP, and nitric oxide (NO) assays, while antidiabetic potential was assessed using α‐glucosidase and α‐amylase inhibitiory assays. Liquid chromatography‐mass spectrometry (LC–MS) was employed to screen and identify the phytochemical compounds in the coffee beans. Additionally, computational studies such as molecular docking and molecular dynamics (MD) simulation were performed to investigate the interactions and stability between the phytochemicals and target enzymes. The results revealed that coffee beans from Burundi exhibited the highest DPPH and FRAP scavenging activities, while Ugandan cofee beans demonstrated the strongest NO scavenging activity. Tanzanian beans showed the most potent inhibition of α‐glucosidase and α‐amylase enzymes. Additionally, Burundian coffee extracts stimulated glucose uptake in yeast cells more effectively than other samples. The LC–MS analysis highlighted that Burundian and Tanzanian beans were rich in phenolic acids, whereas Ugandan beans had the highest caffeine content. Molecular docking and dynamics simulations confirmed strong inhibitory interactions between the identified phytochemicals and target enzymes, supporting their role in antioxidant and antidiabetic activities. The study underscores East African coffee beans' potent antioxidant and antidiabetic properties, attributed to their unique phytochemical profiles. These findings emphasize their potential as functional foods for managing metabolic disorders, providing a scientific basis for their health benefits.

## Graphical abstract

Burundian coffee showed the strongest antioxidant activity (DPPH/FRAP), Ugandan coffee excelled in nitric oxide scavenging, while Tanzanian coffee led in α‐glucosidase/α‐amylase inhibition. LC–MS revealed that phenolic acids dominated in Burundian/Tanzanian beans, whereas Ugandan coffee had the highest caffeine content. Computational studies confirmed the relationship between phytochemical‐enzyme binding and their region‐specific antioxidant/antidiabetic effects, supporting East African coffees as functional foods for metabolic disorders.

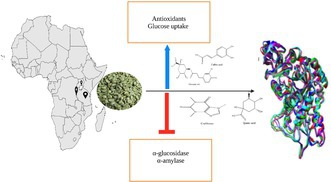

## 1 Introduction

The prevalence of Type 2 Diabetes (T2D) is rapidly increasing in developing nations, with approximately 24 million people in Sub‐Saharan Africa currently affected, according to the International Diabetes Federation (IDF) (IDF 2021). This statistic is expected to increase significantly by 2030, resulting in decreased life expectancy, poorer standards of living, and more expensive healthcare (Abda et al. 2024). Despite extensive research efforts, developing effective and cost‐efficient medications remains a challenge. Therefore, functional foods and nutraceuticals are gaining popularity as a potential alternative for managing T2D (Othman et al. 2025).

Coffee ranks among the most widely consumed beverages globally, with an estimated 40% of the global population drinking it on a regular basis (dePaula and Farah 2019). There are two types of coffee beans: *Coffea arabica* ( *C. arabica* ) and *Coffea robusta* ( *C. robusta* ). Among these, *C. arabica* is one of the most well‐known and popular coffees among consumers worldwide (Deribe 2019). It accounts for approximately 60% of total coffee production worldwide. Its popularity stems from its exceptional taste, aromatic flavor, and unique sensory characteristics. *C. arabica*, originating from 20 countries, from East Asia to Western Latin America (Hameed et al. 2020). In contrast, *C. robusta* is the major coffee bean in many tropical contourites, accounting for 40% of the global coffee bean production (Nadaf et al. 2024).

Recent research has extensively explored coffee beans, focusing on their bioactive components, applications, and preparation methods (Abda et al. 2024). Coffee contains phenolic acids, alkaloids, carbohydrates, proteins, and lipids, all contributing to its bioactive properties. These compounds have significantly mitigated a wide range of diseases, including T2D and its related complications (Gemechu 2020).

The relationship between coffee consumption and T2D has been extensively investigated. Epidemiological evidence suggests that regular coffee intake may lower the risk of developing T2D and its associated conditions. Alongi and Anese (2018) reported that bioactive compounds in coffee, including phenolic acids (chlorogenic) and alkaloids (caffeine), strongly inhibit carbohydrate‐digesting enzymes, implying that they can regulate blood glucose levels. Furthermore, studies suggest that coffee intake may play a role in alleviating key aspects of T2D, such as elevated blood glucose levels, reduced glucose tolerance, insulin resistance, and oxidative stress. Coffee's phytochemical ingredients may be responsible for its health benefits (de Melo Pereira et al. 2020).

There is strong evidence that the pathophysiology of T2D induces oxidative stress brought on by the overproduction of ROS and insulin signaling pathway deficits. However, Nabavi et al. (2017) reported that chlorogenic acid (CGA) lowers oxidative stress by scavenging intracellular ROS, hence boosting the antioxidant defense system. Additionally, CGA has been found to effectively suppress the activity of hydroxyl radicals (OH*), 1,1‐Diphenyl‐2‐picrylhydrazyl (DPPH), 3‐ethylbenothiazoline‐6‐sulfonic acid, and cation radicals in a manner dependent on its concentration.

As extensively documented, coffee beans have potential health benefits, including antioxidants, antidiabetic properties, and protective effects against various metabolic disorders (Mohamed, Erukainure, et al. 2024). However, inadequate research specifically addresses the chemical composition, antioxidant activity, and antidiabetic potential of East African coffee beans compared to South and Central American coffee beans, indicating a substantial research gap. Therefore, investigating these coffees' chemical composition and biological activity would increase their popularity while improving quality control, commercial procedures, and cultivation methods. Accordingly, this study evaluates selected East African coffee beans' chemical compositions and antioxidant and antidiabetic properties. Following a 10‐min roast at 170°C, *C. arabica* from Burundi and Tanzania and *C. robusta* from Uganda were analyzed using in vitro antioxidant and antidiabetic assays, LC–MS, and computational methods. The study is the first to investigate how coffee types ( *C. arabica* and *C. robusta* ) from selected East African origins influence antioxidant and antidiabetic properties. It highlights the unique bioactive profiles shaped by varietal differences, offering valuable insights into the health benefits of region‐specific coffee beans.

## 2 Methods

### 2.1 Coffee Sample Collection

*C. arabica* (from Burundi and Tanzania), as well as *C. robusta* (from Uganda), were collected in July 2024.

### 2.2 Preparation of Coffee Bean Extracts

Coffee beans from Burundi, Uganda, and Tanzania were roasted for 10 min at 170°C before being ground into a fine powder and then defatted using hexane solvent. The coffee powders were then macerated in water for 24 h, followed by decantation, filtration, and freeze‐drying. The obtained extracts were kept at −20°C in sealed containers until further investigation.

### 2.3 Phytochemical Profile of Eastern Africa Coffee Beans

The phytochemical profile was evaluated using total phenolic content (TPC), as the following methods:

### 2.4 Evaluation of Total Phenolic Content (TPC)

Following the approach described by Humadi and Istudor (2009), the TPC of the selected Eastern Africa coffee beans was estimated with the Folin–Ciocalteu method. To prepare the extracts, 50 μL coffee bean extract was mixed with 450 μL of distilled H_2_O, and then 125 μL of Folin–Ciocalteu solvent was added. The combination was put in the dark for 10 min. Following that, 100 μL of a 7% sodium carbonate solution was added, followed by H_2_O to bring the final volume to 500 μL. Absorbance was measured at 750 nm after the reaction was allowed to proceed in darkness for half an hour. A gallic acid standard curve was used to determine the overall phenolic content.

### 2.5 In Vitro Antioxidative Activities Assay

The antioxidant scavenging activities of selected Eastern African coffee beans were evaluated using the following methods: (1) 2,2‐diphenyl‐1‐picrylhydrazyl (DPPH) free radical scavenging assay, (2) ferric reducing antioxidant power (FRAP) assay, and (3) nitric oxide (NO) scavenging assay, as described below:

#### 2.5.1 Evaluate the Free Radical Scavenging Activity

The DPPH free radical method was employed to evaluate the antioxidant activity of selected Eastern African coffee beans, following the protocol previously outlined by Ak and Gülçin (2008). Briefly, equal volumes of coffee bean extracts or gallic acid (as a standard) at various concentrations (30–240 μg/mL) and DPPH solution (0.2 mM in methanol) were mixed. The mixtures were then incubated in the dark at room temperature for 30 min. The assay was performed in triplicate, and the absorbance was measured at 490 nm. The results were calculated according to the following equation:

$$\%\text{DPPH scavenging activity}=\frac{\left({\mathrm{Ab}}_{\text{control}}-{\mathrm{Ab}}_{\text{sample}}\right)}{\left({\mathrm{Ab}}_{\text{control}}\right)}\times 100$$

where, Ab_control_, The absorbance value of the control; Ab_sample_, the absorbance value of the sample.

#### 2.5.2 Ferric (Fe^3+^) Reducing Antioxidant Power (FRAP)

The reducing power of antioxidants in the selected coffee samples was assessed using the FRAP method, adapted from Oyaizu (1986). Briefly, equal volumes (1 mL) of coffee bean extracts at different concentrations (30–240 μg/mL), 1% potassium ferricyanide, and 0.2 M sodium phosphate buffer were mixed and incubated at 50°C for 30 min. Following incubation, 1 mL of distilled water was added, followed by 0.1% ferric chloride (FeCl₃). The absorbance was measured at 700 nm using a spectrophotometer. The reducing power was calculated using the following formula:

$$\begin{array}{c} \%\text{FRAP scavenging activity}=\frac{\left({\mathrm{Ab}}_{\text{control}}-{\mathrm{Ab}}_{\text{sample}}\right)}{\left({\mathrm{Ab}}_{\text{control}}\right)}\times 100 \end{array}$$

where, Ab_control_, the absorbance value of the control; Ab_sample_, the absorbance value of the sample.

#### 2.5.3 Nitric Oxide (NO) Radical Scavenging Activity

The nitric oxide (NO) radical scavenging assay was used to evaluate the capacity of selected coffee beans to inhibit nitric oxide production, following the method reported by Kurian et al. (2022) with minor changes. In brief, equal volumes (50 μL) of coffee bean extracts at varying concentrations (30–240 μg/mL) and sodium nitroprusside (10 mM) were mixed and incubated for 120 min at 37°C. After incubation, an equal volume (50 μL) of Griess reagent was added to the mixture. The assay was performed in triplicate, and the absorbance was measured at 546 nm. The NO scavenging activity of the selected Eastern African coffee beans was calculated using the following equation:

$$\begin{array}{c} \%\text{nitric oxidae}\;\left(\mathrm{NO}\right)\;\text{scavenging activity}=\frac{\left({\mathrm{Ab}}_{\text{control}}-{\mathrm{Ab}}_{\text{sample}}\right)}{\left({\mathrm{Ab}}_{\text{control}}\right)}\times 100 \end{array}$$

where, Ab_control_, the absorbance value of the control; Ab_sample_, the absorbance value of the sample.

### 2.6 In Vitro Carbohydrate Digestive Enzymes Inhibitory Assay

The carbohydrate digestive enzymes inhibitory activities of selected Eastern African coffee beans were evaluated using the following methods: (1) α‐glucosidase and (2) α‐amylase inhibitory assays scavenging assay, as described below:

#### 2.6.1 α‐Glucosidase

The α‐glucosidase inhibitory effects of coffee beans from Burundi, Uganda, and Tanzania were measured by utilizing the outlined method following a modification by Ademiluyi and Oboh (2013). In summary, equal volumes (100 μL) of selected coffee beans in various concentrations (30–240 μg/mL) and α‐glucosidase (2 U/mL) were combined together. The mixture was subjected to an incubation period at 37C^o^ for 10 min. Afterward, 50 μL of p‐Nitrophenyl‐α‐D‐glucopyranoside (pNPG) (5 mM) was added, and the reaction was then incubated for another 20 min at 37°C. All experiments were conducted in triplicate. The α‐glucosidase inhibitory effects of selected coffee beans were calculated using the following equation:

$$\begin{array}{c} \%\alpha -\text{glucosidase inhibitory activity}=\frac{\left({\mathrm{Ab}}_{\text{control}}-{\mathrm{Ab}}_{\text{sample}}\right)}{\left({\mathrm{Ab}}_{\text{control}}\right)}\times 100 \end{array}$$

where, Ab_control_, the absorbance value of the control; Ab_sample_, the absorbance value of the sample.

#### 2.6.2 α‐Amylase Inhibition

The α‐amylase inhibitory effects of coffee beans from Burundi, Uganda, and Tanzania were assessed following a modified version of the assay reported by Shai et al. (2010). In brief, the mixture of 100 μL of coffee bean extracts or acarbose (standard) at concentrations ranging from (30 to 240 μg/mL) and 50 μL of porcine pancreatic amylase (3 U/mL) for 30 min at 37°C. After incubation, 250 μL of starch solution (%1) was introduced, and the reaction proceeded for another incubation for 1 h at 37°C. Then after, 1 mL of dinitrosalicylic acid (DNS) reagent was added, followed by boiling at 100°C for 10 min. The experiment was conducted in triplicate. The α‐amylase inhibitory effects of selected coffee beans were detected at 540 nm, and the calculations were determined utilizing the below equation:

$$\begin{array}{c} \%\alpha -\text{amylase inhibitory activity}=\frac{\left({\mathrm{Ab}}_{\text{control}}-{\mathrm{Ab}}_{\text{sample}}\right)}{\left({\mathrm{Ab}}_{\text{control}}\right)}\times 100 \end{array}$$

where, Ab_control_, the absorbance value of the control; Ab_sample_, the absorbance value of the sample.

### 2.7 Glucose Uptake by Yeast Cells

The impact of coffee beans from Burundi, Uganda, and Tanzania on glucose uptake in yeast cells was evaluated utilizing a modified method based on Erukainure et al. (2019) with minor changes. Briefly, 100 μL of selected coffee extracts at varying concentrations (30–240 μg/mL) and 1 mL of glucose (25 mM) were mixed. The mixture was subjected to an incubation period for 10 min at 37°C. Subsequently, 50 μL of yeast suspension (%1) was induced, vortexed, and incubated for 1 h at 37°C. After incubation, 300 μL of dinitrosalicylate (DNS) reagent was added to the result before being boiled for 10 min. The experiment was conducted in triplicate, and the absorbance was observed at 540 nm. The glucose uptake in yeast cells was computed via the following equation:

$$\begin{array}{c} \%\text{glucose uptake}\;\mathrm{by}\;\text{yeast cells}=1-\frac{\left({\mathrm{Ab}}_{\text{sample}}\;\right)}{\left({\mathrm{Ab}}_{\text{contorl}}\right)}\times 100 \end{array}$$

where, Ab_control_, the absorbance value of the control; Ab_sample_, the absorbance value of the sample.

### 2.8 Determination of Phytochemical Contents of East African Coffee Beans

Phytochemicals in selected Eastern African coffee beans were analyzed using liquid chromatography‐mass spectrometry (LC–MS) (Shimadzu Corporation, Kyoto, Japan). Samples were injected directly via a loop, with an LC–MS runtime of 4 min. The system operated in low‐pressure gradient mode with a PDA sampling frequency of 1.5625 Hz. The mobile phase consisted of H_2_O and CH_3_OH (5:95 ratio) at a flow rate of 0.2000 mL/min. The PDA detector was set to a wavelength range of 190–800 nm and a cell temperature of 40°C. The acquisition was performed in scan mode with positive polarity, an event duration of 1.00 s, a detector voltage of +1.00 kV, and a scan range of m/z 50.00–1700.00 at a speed of 1.667 u/s. Coffee beans' phytochemicals were identified by matching retention times and mass spectra with the previous literature and NIST database.

### 2.9 Computational Study

The computational study was conducted via (1) molecular docking and (2) molecular dynamics (MD) simulations, as described below:

#### 2.9.1 Molecular Docking

The identified compounds from Burundi, Uganda, and Tanzania coffee beans were analyzed through molecular docking to assess their binding affinities at the active sites of α‐glucosidase and α‐amylase. The 3D structures of α‐glucosidase (PDB ID: 3L4Y) (Sim et al. 2010) and α‐amylase (PDB ID: 3O9N) (Duong 2020) were retrieved from the Protein Data Bank (PDB) and prepared using UCSF Chimera software [23]. The compounds were optimized for correct hybridization states and molecular geometry using MarvinSketch 6.2.1, Molegro Molecular Viewer (MMV), and ChemAxon. Docking simulations were performed using the Desmond software (Schrödinger 2023–2) with the OPLS‐2005 force field. The resulting compound‐enzyme complexes were visualized and analyzed using BIOVIA Discovery Studio Visualizer.

#### 2.9.2 Molecular Dynamic (MD) Simulation

Molecular dynamics (MD) simulations were performed using Desmond software (Schrödinger 2023–2) to evaluate the structural stability and dynamic behavior of apo and docked ligand‐complexes involving coffee‐derived phytochemicals with α‐glucosidase and α‐amylase. The OPLS‐2005 force field and an explicit solvent model with SPC water molecules were employed (Chow et al. 2008; Martyna et al. 1994). Sodium ions (Na^+^) were added to neutralize the system, and NaCl (0.15 M) solution was incorporated to replicate the physiological environment. System equilibration was conducted in two stages: 200 ps under the NVT ensemble and 12 ps under the NPT ensemble, utilizing the Nosé–Hoover chain coupling approach. The simulation temperature was set at 27°C with a relaxation period of 1.0 ps, while pressure was regulated at 1 bar using the Martyna–Tuckerman–Klein chain coupling scheme with a 2 ps relaxation time. Long‐range electrostatic interactions were calculated using the particle mesh Ewald method, with a Coulomb cutoff set at 9 Å. Bonded forces were estimated using the RESPA integrator with a 2 fs time step. System stability was assessed through Root Mean Square Deviation (RMSD), Radius of Gyration (RoG), Root Mean Square Fluctuation (RMSF), and Solvent‐Accessible Surface Area (SASA). A clustering analysis of RMSD trajectories over a 100 ns period was performed using the Desmond trajectory clustering module, with a clustering frequency of 20 and 10 potential clusters (Martyna et al. 1994; Mukund et al. 2019). All simulations were executed remotely at the Centre for High Performance Computing (CHPC) in Cape Town.

##### 2.9.2.1 MM‐GBSA Analysis

The ligand‐protein complexes binding affinities were calculated using the Molecular Mechanics Generalized Born Surface Area (MM‐GBSA) method. The ΔG_bind_ values were calculated from simulation trajectories using the *thermal_mmgbsa.py* Python script, incorporating the VSGB solvation model and the OPLS5 force field. Calculations were performed on the final 50 frames, with a sampling interval of one step. The ΔG_bind_ was determined by summing the contributions of various energy components, such as Coulombic interactions, hydrogen bonds, van der Waals forces, lipophilicity effects, and solvation energies of the ligand‐protein system (Mukund et al. 2019). The equation for ΔG_bind_ is as follows:

$$\begin{array}{c} {\mathrm{\Delta G}}_{\text{bind}}=\;G_{\mathrm{MM}}+\;G_{\text{Solv}}-\;G_{\mathrm{SA}} \end{array}$$

where, ΔG_bind_, the free binding energy; ΔG_MM_, the difference between the free energy of the ligand‐protein complex and the combined energies of the isolated protein and ligand; ΔG_Solv_, the difference in solvation energies between the ligand‐receptor complex and the sum of the solvation energies of the unbound receptor and ligand; ΔG_SA_, the change in surface area energies between the protein and ligand.

### 2.10 Statistics

All data were presented as mean ± SD. The statistical analysis used a one‐way analysis of variance (ANOVA), with significance set at *p* < 0.05. The data were analyzed using IBM's Statistical Package for the Social Sciences (SPSS) for Windows, version 27.0 (IBM Corp., Armonk, NY, USA).

## 3 Results and Discussion

### 3.1 Total Phenolic Content (TPC)

**FIGURE 1:**
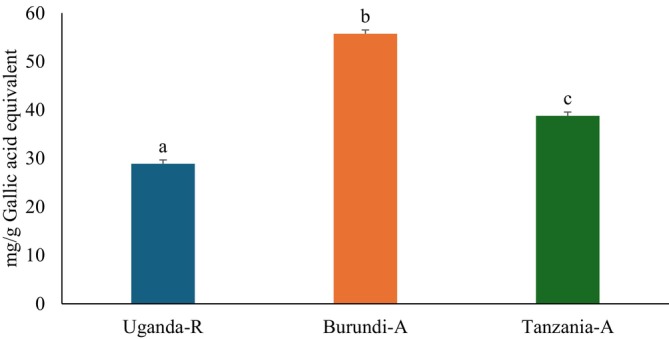
Total phenolic contents of Uganda, Burundi, and Tanzania coffee beans. Values are presented as mean ± SD of triplicate determinations. (a–c) Bars with different letters above the bars are significantly different from each other (*p* < 0.05). Burundi‐A: Burundian Arabica coffee; QE, Quercetin equivalent; Tanzania‐A, Tanzanian Arabica coffee; Uganda‐R, Ugandan Robusta coffee.

The consumption of phenolic phytochemicals plays a crucial role in diabetes management since they can reduce blood glucose levels and oxidative stress, and inhibit other key processes related to carbohydrate metabolism (de Paulo Farias et al. 2021). This study demonstrated that Burundi coffee beans had the highest TPC at 55.72 ± 0.87 mg GAE/g, followed by Tanzanian coffee beans at 38.00 ± 1.61 mg GAE/g. Ugandan coffee beans had the lowest TPC at 28.90 ± 0.48 mg GAE/g, as shown in Figure 1. Furthermore, *C. arabica* exhibited more TPC than *C. robusta* , presumably attributable to the increased concentration of phenolic acids in *C. arabica* . Limited studies have been conducted on the phytochemical composition of selected East African coffee beans. However, the current study confirms that these beans have a high TPC, indicating excellent pharmacological properties.

### 3.2 Antioxidant Scavenging Activities

**FIGURE 2:**
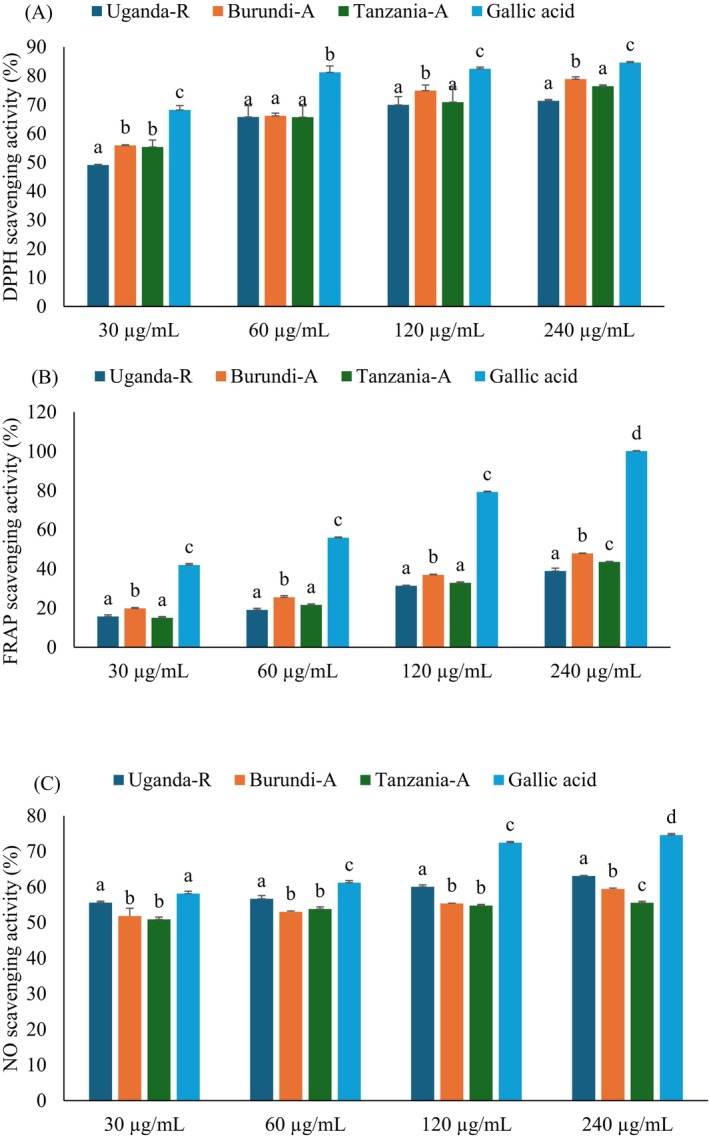
(A) DPPH, (B) FRAP, and (C) Nitric oxide scavenging activities of Uganda, Burundi, and Tanzania coffee bean extracts. Values are presented as mean ± SD of triplicate determinations. (a–d) Bars with different letters over the bars for a given concentration are significantly different from each other (*p* < 0.05). Burundi‐A, Burundian Arabica coffee; Tanzania‐A, Tanzanian Arabica coffee; Uganda‐R, Ugandan Robusta coffee.

Antioxidants protect the body's cellular structure from oxidative stress caused by ROS (Adwas et al. 2019). The main role of antioxidants is to mitigate oxidative damage, significantly preventing various diseases, including T2D and its associated complications (Burgos‐Morón et al. 2019). The antioxidant scavenging activities of Burundi, Uganda, and Tanzanian coffee beans were evaluated through DPPH, FRAP, and NO, as presented in Figure 2, and their IC_50_ values are shown in Table 1. The findings revealed that the coffee beans under investigation demonstrated dose‐dependent inhibition, with statistically marginal differences (*p* < 0.05) observed across all samples at elevated concentrations in various antioxidant assays. Notably, Burundi coffee beans displayed significantly enhanced scavenging activity (*p* < 0.05) in a dose‐dependent manner for both DPPH and FRAP assays, whereas Ugandan coffee beans exhibited the highest activity (*p* < 0.05) in the NO assay. The IC_50_ values for Burundi coffee beans were 24.74 μg/mL for DPPH and 309.83 μg/mL for FRAP, while Ugandan coffee beans recorded an IC_50_ value of 44.49 μg/mL for NO, as detailed in Table 1. These outcomes underscore the high antioxidant potential of East African coffee, particularly *C. arabica* , which may be attributed to its elevated TPC (Jeszka‐Skowron et al. 2016). The reproducible antioxidant efficacy observed across all analyzed coffee samples corroborates the extensively documented function of coffee as a nutritionally valuable source of redox‐active phytochemicals capable of mitigating oxidative stress (Samsonowicz et al. 2019; Yohannis et al. 2024). The study highlights the potential of East African coffee beans to enhance antioxidant activity, emphasizing their nutritional and functional significance.

**TABLE 1:** IC_50_ values (μg/mL) for selected East African coffee beans' antioxidant and antidiabetic activities.

Biological activities	Uganda (Robusta coffee)	Burundi (Arabica coffee)	Tanzania (Arabica coffee)	Gallic acid	Acarbose	Metformin
DPPH	31.98	24.74	27.09	15.54	—	—
FRAP	1157.03	309.83	543.88	12.64	—	—
NO	44.49	59.90	66.65	27.68		
α‐glucosidase	100.17	54.68	52.70	—	41.80	
α‐amylase	36.34	32.89	31.93	—	21.03	
Glucose uptake by yeast cells	247.61	132.76	201.54			73.06

*Note:* All values are expressed as μg/mL.

Abbreviations: —, not applicable; DPPH, 2,2‐diphenyl‐1‐picrylhydrazyl; FRAP, ferric reducing antioxidant power; NO, nitric oxide.

### 3.3 Carbohydrate Digestive Enzymes Inhibitory Activity

**FIGURE 3:**
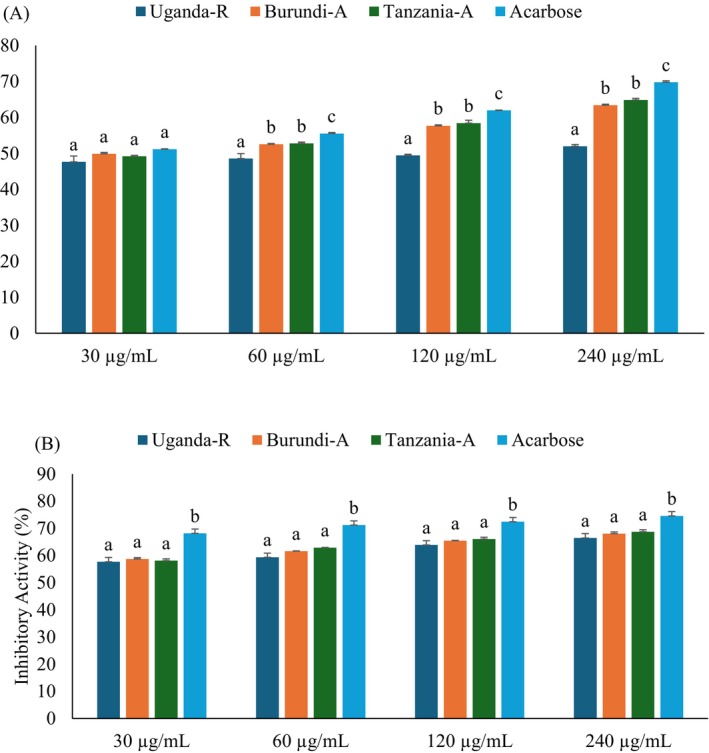
(A) α‐glucosidase and (B) α‐amylase inhibitory activities of Ugandan, Burundian, and Tanzanian coffee bean extracts. Values are presented as mean ± SD of triplicate. (a–c) Bars with different letters over the bars for a given concentration are significantly different from each other (*p* < 0.05). Burundi‐A, Burundian Arabica coffee; Tanzania‐A, Tanzanian Arabica coffee; Uganda‐R, Ugandan Robusta coffee.

Intestinal α‐glucosidase and pancreatic α‐amylase play critical roles in carbohydrate metabolism by breaking down dietary starches into absorbable monosaccharides (Li et al. 2022). Both enzymes have been recognized as therapeutic candidates for managing postprandial hyperglycemia, a first metabolic abnormality in T2D (Sottero et al. 2015). Inhibiting α‐glucosidase and α‐amylase diminishes carbohydrate absorption, mitigates postprandial glucose surges, and presents a promising treatment strategy for T2D management (Kashtoh and Baek 2022). In the present study, the carbohydrate digestive enzyme inhibitors such as α‐glucosidase and α‐amylase assays were employed to evaluate the in vitro anti‐diabetic properties of Burundian, Ugandan, and Tanzanian coffee beans, as seen in Figure 3A,B and Table 1. Regarding the results, all studied coffees showed dose‐dependent inhibitory activity against α‐glucosidase and α‐amylase. In comparison between the studied coffee beans, Tanzanian coffee beans displayed a higher significant inhibitory action (*p* < 0.05) against α‐glucosidase and α‐amylase, with IC_50_ values of 52.70 μg/mL and 31.93 μg/mL, respectively. This was followed by Burundian coffee beans, while the Ugandan coffee beans showed the lowest inhibitory action against carbohydrate digestive enzymes. Furthermore, *C. arabica* performed superior antidiabetic properties to *C. robusta*, likely due to the high TPC, consistent with previous literature that reported coffee polyphenols, such as CGA, exhibited significant inhibitory effects on carbohydrate‐digesting enzymes (Alongi et al. 2021; Li et al. 2025). However, these findings represent the first comprehensive report on the comparative enzyme inhibitory properties of the studied coffee beans, highlighting their potential antidiabetic effects.

### 3.4 Glucose Uptake by Yeast Cells

**FIGURE 4:**
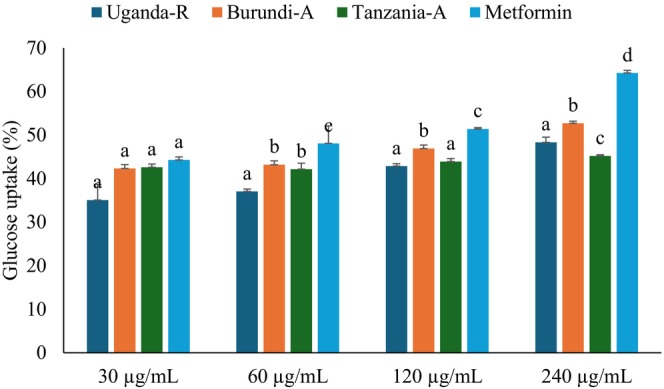
Effect of Uganda, Burundi, and Tanzania coffee bean extracts on glucose uptake in yeast cells. Values are presented as mean ± SD of triplicate determinations. (a–d) Bars with different letters over the bars for a given concentration are significantly different from each other (*p* < 0.05). Burundi‐A, Burundian Arabica coffee; Tanzania‐A, Tanzanian Arabica coffee; Uganda‐R, Ugandan Robusta coffee.

The glucose‐regulation system, which controls gluconeogenesis and alternate sugar catabolism, has been widely studied in research (Remesar and Alemany 2020). A key strategy is to identify natural or synthetic antidiabetic drugs by assessing their ability to increase glucose uptake, often employing yeast cells as a model organism (Zimmermann et al. 2018). In this study, yeast cells have been employed to evaluate the glucose uptake, as illustrated in Figure 4 and Table 1. All studied coffee beans showed dose‐dependent activity on glucose uptake, with Burundian coffee demonstrating the highest activity, followed by Tanzanian coffee beans. The IC_50_ values of Burundian and Tanzanian coffee beans were (132.76 μg/mL) and (201.54 μg/mL), respectively, as shown in Table 1. These coffee beans' observed enhancement in glucose uptake may be attributed to their unique phytochemical compositions (Singh et al. 2023). While research on East African coffee beans, such as those from Uganda, Tanzania, and Burundi, is limited, the findings demonstrate that they can bind glucose and accelerate its translocation across cell membranes, highlighting their antidiabetic properties.

### 3.5 LC–MS Analysis

The liquid chromatography‐mass spectrometry (LC–MS) was utilized to screen and characterize bioactive compounds present in selected East African coffee beans. Both deprotonated [M‐H]^−^ and protonated [M + H]^+^ ion modes were used for spectral analysis (Mohamed, Olofinsan, et al. 2024). Compound identification was based on retention time (Rt) and comparison with MS data from literature and relevant databases. A total of 8 bioactive compounds were identified (Figure S1; Table 2). *C. arabica* demonstrated a significantly higher concentration of phenolic acids, while *C. robusta* was characterized by elevated levels of caffeine. These findings are consistent with earlier research reported by Jeszka‐Skowron et al. (2016), further validating the observed trends in the compositional differences between the two coffee species. Additionally, Burundian coffee beans were identified as having the highest CGA content, while Ugandan coffee beans showed the lowest quinic acid abundance, Table 2.

**TABLE 2:** Characterization of the compounds found in the different extracts of Kenyan, Uganda, Burundi, and Tanzania coffee beans.

NO.	T_R_ (min)	Ionization	*m/z* (base peak)	Assigned identification	% abundant		
					Uganda (Robusta coffee)	Burundi (Arabica coffee)	Tanzania (Arabica coffee)
1	6.62	[M‐H]^−^	353	Chlorogenic acid	6.62	17.10	14.15
2	8.36	[M + H]^+^	195	Caffeine	15.22	5.67	0.14
3	4.90	[M‐H]^−^	197	Caffeic acid	0.04	2.07	0.94
4	2.31	[M‐H]^−^	191	Quinic acid	0.22	0.10	4.6
5	8.38	[M‐H]^−^	188	2,6‐di‐tert‐butylphenol	nd	0.98	1.06
6	7.87	[M‐H]^−^	205	Methanone, bis(4‐phenoxyphenyl)—	0.06	2.24	3.17
7	23.46	[M‐H]^−^	337	3‐p‐coumaroylquinic acid	nd	0.98	1.06
8	6.44	[M‐H]^−^	153	Protocatechuic acid	0.14	nd	0.13

Abbreviations: nd, not detected; T_R_, Retention time.

### 3.6 Molecular Docking Results

Computational techniques have become indispensable in drug development (Sliwoski et al. 2014). These techniques, such as molecular docking, serve to comprehend the interaction between ligands and receptors, enabling the prediction of inhibitors of proteins involved in metabolic disorders, such as T2D (Agu et al. 2023). In this section, the identified compounds from Ugandan, Burundian, and Tanzanian coffee beans (Table 3) and acarbose (standard) have been subjected to molecular docking to evaluate their interaction with the α‐glucosidase and α‐amylase. Table 3 and Figure S2 display their interactions with pocket sites and free binding scores. Among the identified compounds in selected East African coffee beans, Methanone, bis‐(4‐phenoxyphenyl)‐ exhibited the strongest binding affinities with α‐glucosidase and α‐amylase, with binding energies of −5.54 kcal/mol and − 6.21 kcal/mol, respectively. In contrast, Protocatechuic acid showed the weakest interactions, with binding energies of −4.26 kcal/mol and − 3.72 kcal/mol for α‐glucosidase and α‐amylase, respectively.

**TABLE 3:** Free binding energy interaction of selected East African coffee compounds with the target proteins.

Compounds	α‐glucosidase (kcal/mol)	α‐amylase (kcal/mol)
Chlorogenic acid	−5.49	−5.74
Caffeine	−5.18	−4.06
Caffeic acid	−4.67	−4.23
Quinic acid	−4.59	−3.98
2,6‐di‐tert‐butylphenol	−5.05	−4.73
Methanone, bis(4‐phenoxyphenyl)—	−5.54	−6.21
3‐p‐coumaroylquinic acid	−5.36	−5.39
Protocatechuic acid	−4.26	−3.72
Acarbose	−10.36	−7.69

*Note:* All values are expressed as kcal/mol.

Methanone, bis(4‐phenoxyphenyl)‐ formed 5 hydrogen bonds (H‐acceptor) and 6 ionic bonds with key residues at the active site of α‐glucosidase, including ARG200, HIS332, ASP333, ARG400, and ARG200. For α‐amylase, it demonstrated hydrogen bond (H‐acceptor) and pi‐H interactions with ARG232 and MET300 at the active site. Further details are provided in Figure S2 and Table 3. These results indicate that the selected East African coffee bean compounds show robust binding interactions with the carbohydrate enzymes, suggesting their potential application in the formulation of natural antidiabetic treatments.

### 3.7 Molecular Dynamics (MD) Simulations

**FIGURE 5:**
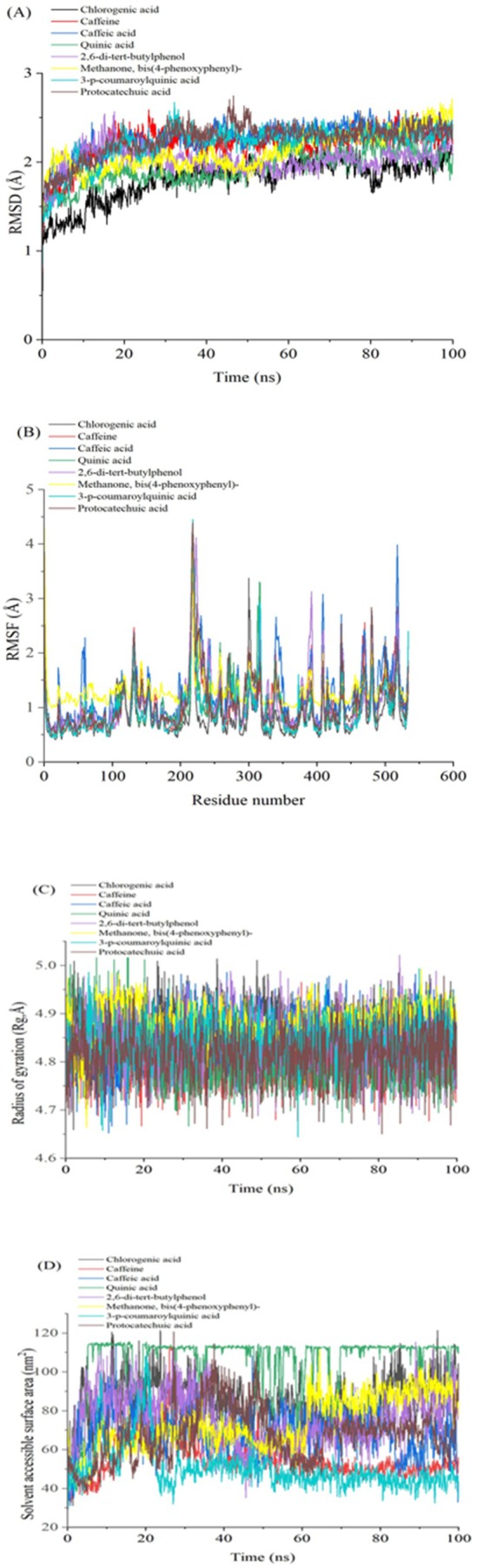
Plot displaying (A) RMSD, (B) RMSF, (C) RoG, and (D) SASA for identified compounds from East African coffee beans with α‐glucosidase over the 100 ns MD simulation time. RMSD, root mean square deviation; RMSF, root mean square fluctuation; RoG, radius of gyration; SASA, solvent‐accessible surface area.

**FIGURE 6:**
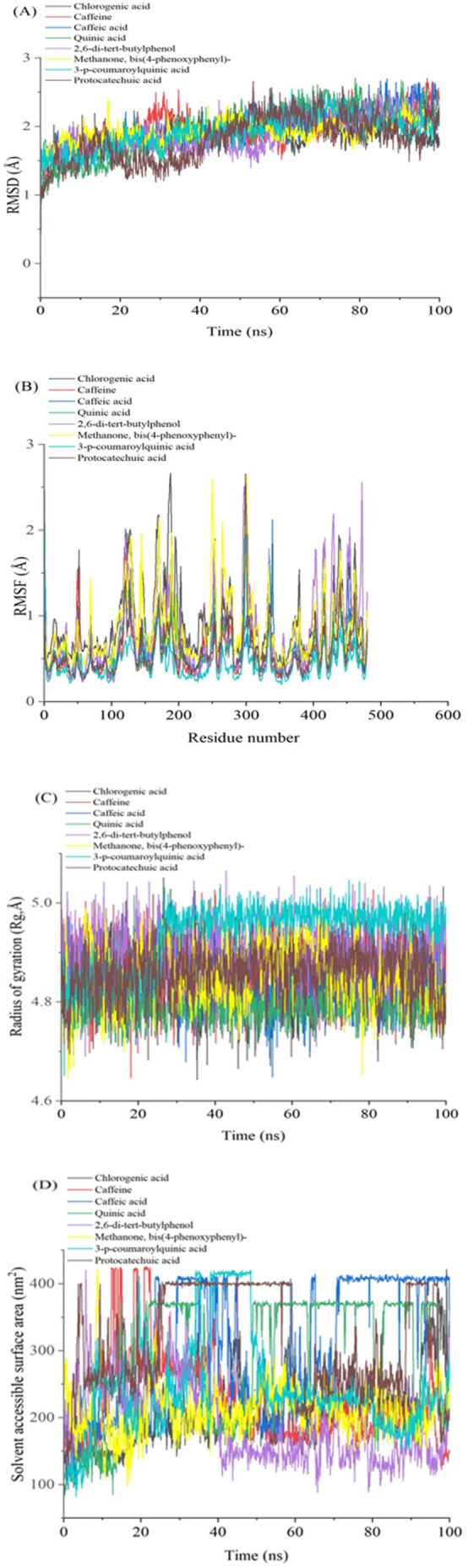
Plots displaying (A) RMSD, (B) RMSF, (C) RoG, and (D) SASA for identified compounds from East African coffee beans with α‐amylase over the 100 ns MD simulation time. RMSD, root mean square deviation; RMSF, root mean square fluctuation; RoG, radius of gyration; SASA, solvent‐accessible surface area.

Molecular dynamics (MD) simulations were performed to evaluate bioactive compounds' inhibition potential and stability from selected East African coffee beans in complex with α‐glucosidase and α‐amylase. System stability was assessed using Root‐Mean‐Square Deviation (RMSD) analysis over a 100 ns simulation period. Notably, all compounds exhibited low RMSD values, indicating high structural stability. Methanone, bis(4‐phenoxyphenyl)‐ demonstrated the highest stability with α‐glucosidase, recording an average RMSD of 1.62 Å (Figure 5A; Table 4). Similarly, 2,6‐di‐tert‐butylphenol showed the lowest RMSD value of 1.55 Å against α‐amylase, further confirming strong stability (Figure 6A; Table 4). These consistently low RMSD values across all compounds suggest robust binding and structural integrity, highlighting their potential as effective enzyme inhibitors.

**TABLE 4:** RMSD, RMSF, RoG, and SASA profiles of identified compounds from selected East African coffee beans bound to α‐glucosidase and α‐amylase.

Compounds	RMSD (Å)	RMSF (Å)	RoG (Å)	SASA (Å^2^)
α‐glucosidase				
Chlorogenic acid	1.80	0.84	4.86	84.90
Caffeine	2.05	0.93	3.03	113.28
Caffeic acid	2.14	0.87	3.12	56.06
Quinic acid	1.75	0.91	2.42	300.42
2,6‐di‐tert‐butylphenol	1.85	0.87	3.39	45.76
Methanone, bis(4‐phenoxyphenyl)—	1.62	0.85	5.53	96.54
3‐p‐coumaroylquinic acid	2.07	0.86	4.76	83.40
Protocatechuic acid	1.75	0.86	2.56	62.19
α‐amylase				
Chlorogenic acid	1.84	0.92	4.84	206.13
Caffeine	1.97	0.92	173.79	3.03
Caffeic acid	1.62	0.90	267.97	3.10
Quinic acid	1.93	1.04	258.52	2.41
2,6‐di‐tert‐butylphenol	1.55	0.85	190.06	3.39
Methanone, bis(4‐phenoxyphenyl)—	1.58	0.84	241.44	5.38
3‐p‐coumaroylquinic acid	1.91	0.88	344.25	4.70
Protocatechuic acid	1.82	0.90	231.53	2.55

Abbreviations: RMSD, root‐mean‐square deviation; RMSF, root‐mean‐square fluctuation; RoG, radius of gyration; SASA, solvent accessible surface area.

The stability of molecular dynamics (MD) simulations is a critical factor, as it determines the reliability and validity of the results obtained from such computational studies (De Vivo et al. 2016). Root mean square fluctuation (RMSF) results are useful for assessing residue flexibility in protein‐ligand complexes, providing insights into their dynamic behavior over the simulation period (Khan et al. 2017). For α‐glucosidase, CGA exhibited the lowest fluctuations with an average RMSF of 0.84 Å, indicating rigid binding, while caffeine showed the highest fluctuations with an average of 0.93 Å (Figure 5B; Table 4), suggesting greater flexibility. Notably, significant fluctuations were observed in specific residue regions, particularly between positions 210–125 and 520, indicating areas of potential conformational flexibility within the α‐glucosidase structure. For α‐amylase, Methanone, bis(4‐phenoxyphenyl)‐ demonstrated the lowest fluctuations with an average RMSF of 0.84 Å, indicating stable binding, while quinic acid exhibited the highest fluctuations with an average of 1.04 Å (Figure 6B; Table 4). Key fluctuating regions in α‐amylase were observed at residues 110–135, 170–175, 180, 200, 250, 300, and 480. These fluctuations may arise from loop rearrangements or conformational changes in secondary structures upon ligand binding, suggesting dynamic flexibility in these regions (Papaleo et al. 2016). Overall, the RMSF analysis reveals that the flexibility of residues varies significantly across compounds and enzymes, with specific regions showing higher conformational variability, which may influence ligand binding and protein dynamics.

The radius of gyration (RoG) serves as a key parameter for assessing the compactness of protein structures, where reduced RoG values correlate with tighter molecular packing and a more condensed conformation (Sarthak et al. 2023). In this study, RoG analysis was used to assess the structural dynamics of α‐glucosidase and α‐amylase complexes with selected East African coffee bioactive compounds. Throughout the 100 ns simulation, the RoG profiles for both enzyme complexes remained stable, suggesting that the protein structures achieved a well‐folded and converged state Table 4. For the α‐glucosidase complexes, quinic acid exhibited the lowest RoG value of 2.42 Å (Figure 5C; Table 4), while for α‐amylase, quinic acid also showed the lowest RoG value of 2.41 Å (Figure 6C; Table 4). These significantly lower RoG values suggest a more compact orientation of the protein‐ligand complexes, likely due to tighter packing of residues upon ligand binding (Evoli et al. 2014). Overall, the RoG analysis provides quantitative evidence of structural changes induced by ligand binding, highlighting the impact of these compounds on the compactness and conformational dynamics of α‐glucosidase and α‐amylase.

The solvent‐accessible surface area (SASA) was used to assess the interaction between the protein surface and solvent atoms upon ligand binding, as illustrated in Figure 4. For α‐glucosidase complexes, quinic acid exhibited the highest SASA value of 300.42 Å^2^, indicating a large surface area exposed to the solvent. In contrast, caffeic acid showed the lowest SASA value of 56.06 Å^2^, suggesting a more compact or less solvent‐exposed structure (Figure 5D; Table 4). For α‐amylase complexes, quinic acid again displayed the highest SASA value of 258.52 Å^2^, while caffeine had the lowest value of 173.79 Å^2^ (Figure 6D; Table 4). These SASA values highlight differences in solvent accessibility and structural compactness among the compounds, providing insights into how ligand binding influences protein‐solvent interactions and overall conformational dynamics (Salifu et al. 2023).

### 3.8 MM–GBSA Calculations

The MM‐GBSA approach, based on molecular dynamics (MD) simulation trajectories, was used to calculate the binding free energy and its contributing energy components for phytochemicals from selected East African coffee beans bound to α‐glucosidase and α‐amylase. As shown in Table 5, Methanone, bis(4‐phenoxyphenyl)‐ exhibited the strongest binding affinities, with ΔG_bind_ values of −53.94 ± 4.87 kcal/mol for α‐glucosidase and − 44.26 ± 4.97 kcal/mol for α‐amylase. The stability of these complexes was driven by significant contributions from van der Waals (ΔG_bindvdW_), electrostatic (ΔG_bindHbond_), lipophilic (ΔG_bindLipo_), and solvation (ΔG_bindSolvGB_) energies. Among the studied compounds, Methanone, bis(4‐phenoxyphenyl)‐ displayed the highest binding affinity, followed by CGA (−46.90 ± 7.65 kcal/mol for α‐glucosidase and − 40.56 ± 9.03 kcal/mol for α‐amylase), while Quinic acid showed the weakest binding (−2.74 ± 6.80 kcal/mol for α‐glucosidase and − 7.42 ± 7.03 kcal/mol for α‐amylase), as seen in Table 5. These results highlight the potential of the studied coffee beans as a strong inhibitor of both enzymes, with van der Waals and electrostatic interactions playing key roles in stabilizing the complexes.

**TABLE 5:** MM/GBSA‐based binding free energy profile of selected East African coffee beans bound to α‐glucosidase and α‐amylase.

Systems	Energy components (kcal/mol)						
	ΔG_bind_	ΔG_bindCoulomb_	ΔG_bindCovalent_	ΔG_bindHbond_	ΔG_bindLipo_	ΔG_bindSolvGB_	ΔG_bindvdW_
α‐glucosidase							
Chlorogenic acid	−46.90 ± 7.65	54.36 ± 27.90	2.28 ± 1.76	−3.04 ± 0.47	−15.59 ± 1.19	−41.04 ± 27.30	−43.40 ± 2.58
Caffeine	−23.85 ± 7.12	−4.18 ± 3.31	0.31 ± 0.27	−0.07 ± 0.13	−5.85 ± 1.37	10.95 ± 6.62	−23.06 ± 3.63
Caffeic acid	−18.91 ± 3.96	69.97 ± 23.38	2.32 ± 1.13	−2.39 ± 0.67	−10.47 ± 1.04	−22.59 ± 1.84	2.38 ± 1.00
Quinic acid	−2.74 ± 6.80	18.37 ± 31.46	0.149 ± 0.55	−0.381 ± 0.68	−1.06 ± 1.87	−16.01 ± 28.81	−3.80 ± 6.39
2,6‐di‐tert‐butylphenol	−40.29 ± 5.44	−2.80 ± 1.163	0.97 ± 0.97	−0.03 ± 0.17	−19.67 ± 1.85	18.00 ± 2.47	−33.33 ± 2.15
Methanone, bis(4‐phenoxyphenyl)—	−53.94 ± 4.87	−6.80 ± 3.97	4.54 ± 2.49	−0.31 ± 0.30	−29.34 ± 2.38	33.65 ± 8.01	−51.63 ± 4.86
3‐p‐coumaroylquinic acid	−41.81 ± 5.40	65.44 ± 20.25	2.40 ± 1.39	−2.04 ± 0.73	−16.73 ± 1.74	−41.99 ± 4.62	2.28 ± 1.22
Protocatechuic acid	−14.19 ± 4.10	82.41 ± 21.27	1.09 ± 0.99	−2.47 ± 0.54	−7.11 ± 1.17	−69.45 ± 19.63	−18.44 ± 3.50
α‐amylase							
Chlorogenic acid	−40.56 ± 9.03	14.25 ± 9.49	3.36 ± 1.38	−3.39 ± 0.73	−12.40 ± 2.68	−9.55 ± 7.52	−31.35 ± 4.77
Caffeine	−18.97 ± 10.19	−4.78 ± 3.81	0.25 ± 0.32	−0.24 ± 0.30	−2.56 ± 1.57	7.14 ± 3.89	−16.14 ± 8.40
Caffeic acid	−10.06 ± 7.52	8.163 ± 20.48	0.90 ± 1.04	−0.91 ± 0.84	−3.07 ± 2.83	−5.08 ± 17.82	−9.60 ± 6.97
Quinic acid	−7.42 ± 7.03	3.23 ± 18.94	0.39 ± 0.80	−1.15 ± 1.20	0.00	−1.72 ± 16.51	−6.75 ± 6.41
2,6‐di‐tert‐butylphenol	−22.62 ± 4.62	−2.42 ± 2.37	0.56 ± 0.93	−0.11 ± 0.22	−10.19 ± 2.93	10.79 ± 4.19	−20.51 ± 3.83
Methanone, bis(4‐phenoxyphenyl)—	−44.26 ± 4.97	−4.39 ± 2.58	5.06 ± 2.26	−0.31 ± 0.28	−20.77 ± 2.17	19.43 ± 2.47	−39.95 ± 3.17
3‐p‐coumaroylquinic acid	−21.92 ± 7.95	9.318 ± 13.24	2.03 ± 1.70	−1.64 ± 0.81	−6.36 ± 2.89	−4.58 ± 12.33	−19.80 ± 6.80
Protocatechuic acid	−11.04 ± 5.61	16.88 ± 19.22	1.05 ± 1.23	−1.65 ± 0.98	−2.94 ± 1.66	−14.06 ± 18.00	−9.86 ± 4.72

Abbreviation: MM‐GBSA, molecular mechanics‐generalized born surface area.

## 4 Conclusion

This study provides the first comprehensive comparative analysis of the antioxidant, antidiabetic, and phytochemical properties of East African coffee beans, evaluating *C. robusta* (Uganda) and *C. arabica* (Burundi, Tanzania). Findings revealed that Burundian *C. arabica* exhibited the highest antioxidant activity, while Tanzanian *C. arabica* showed the most potent inhibition of carbohydrate‐digesting enzymes, underscoring its antidiabetic potential. Phytochemical profiling identified distinct compositional differences: *C. arabica* was richer in phenolic acids, whereas *C. robusta* contained higher caffeine levels. Computational analyses corroborated these results, demonstrating strong binding interactions between coffee‐derived compounds and key digestive enzymes, further supporting their mechanistic role in antidiabetic effects. Collectively, these findings highlight East African coffee as a promising source of bioactive compounds for functional foods targeting metabolic disorders. Future studies should prioritize in vivo validation and isolation of active constituents to assess their therapeutic applications.

## Author Contributions

**Almahi I. Mohamed:** data curation (equal), formal analysis (equal), investigation (equal), methodology (equal), software (equal), validation (equal), visualization (equal), writing – original draft (equal), writing – review and editing (equal). **Ochuko L. Erukainure:** validation (equal), visualization (equal), writing – original draft (equal), writing – review and editing (equal). **Huda Ismail:** data curation (equal), methodology (equal), writing – original draft (equal), writing – review and editing (equal). **Md. Shahidul Islam:** conceptualization (equal), funding acquisition (equal), project administration (equal), supervision (equal), validation (equal), visualization (equal), writing – review and editing (equal).

## Conflicts of Interest

The authors declare no conflicts of interest.

## Supporting information


**Figure S1.** Identified compounds from Uganda, Burundi, and Tanzania coffee beans using LC‐MC analysis.
**Figure S2.** The 3D and 2D interaction between Methanone, bis(4‐phenoxyphenyl)‐ and α‐glucosidase and α‐amylase.

## Data Availability

All data are presented in the article.
