# Supplementary material for: Exploring the Chemical Profile, Antioxidants, and Anti‐Diabetic Properties of Coffee Beans From Selected East African Countries: A Comparative In Vitro and Computational Study
Source: Food Sci Nutr. 2025 Jul 14;13(7):e70527. doi: 10.1002/fsn3.70527 (PMC12259390; doi:10.1002/fsn3.70527)
Supplement: Supplementary file 1 — Figure S1. Identified compounds from Uganda, Burundi, and Tanzania coffee beans using LC‐MC analysis. Figure S2. The 3D and 2D interaction between Methanone, bis(4‐phenoxyphenyl)‐ and α‐glucosidase and α‐amylase. [file FSN3-13-e70527-s001.docx]

|  |  |
| --- | --- |
|  |  |
|  |  |
|  |  |

**Fig. S1.** Identified compounds from Uganda, Burundi, and Tanzania coffee beans using LC-MC analysis.


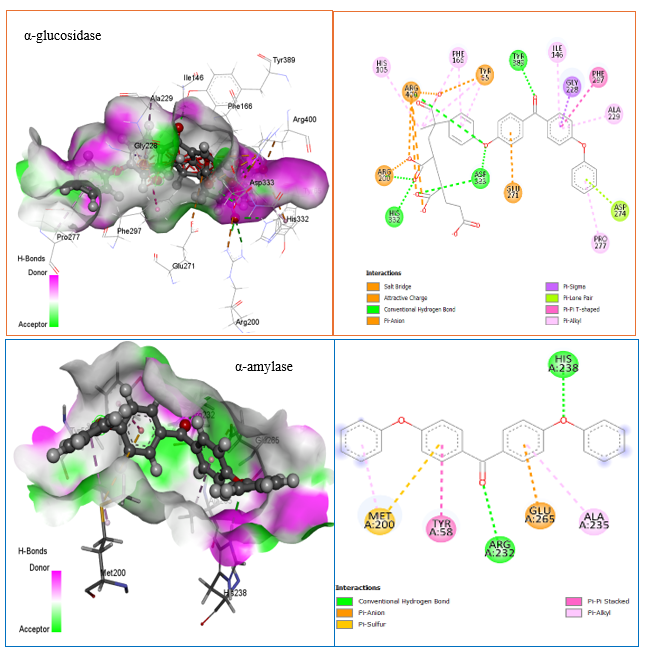


**Fig. S2.** The 3D and 2D interaction between Methanone, bis(4-phenoxyphenyl)- and α-glucosidase and α-amylase.
